# Networks to strengthen community social capital for suicide prevention in regional Australia: the LifeSpan suicide prevention initiative

**DOI:** 10.1186/s13033-022-00524-z

**Published:** 2022-02-07

**Authors:** Janet C. Long, Colum Ruane, Louise A. Ellis, Rebecca Lake, Anneke Le Roux, Luke Testa, Fiona Shand, Michelle Torok, Yvonne Zurynski

**Affiliations:** 1 Australian Institute of Health Innovation, Macquarie University, North Ryde, Level 6, 75 Talavera Rd, Sydney, NSW 2109 Australia; 2 NHMRC Partnership Centre in Health System Sustainability, Australian Institute of Health Innovation, Macquarie University, Sydney, NSW Australia; 3 Black Dog Institute, University of New South Wales, Sydney, Australia

**Keywords:** Social network analysis, Suicide prevention, Social capital, Integrated care

## Abstract

**Introduction:**

Mental health services are fragmented in Australia leading to a priority being placed on whole-of-community approaches and integration. We describe the LifeSpan suicide prevention intervention developed by the Black Dog Institute that draws upon nine evidence-based community-wide strategies. We examined the suicide prevention Collaborative group at each site. We evaluated how the social capital of the community and service providers changed, and how the brokerage roles of the Collaborative affected integration of effort.

**Methods:**

This was a two phase, explanatory mixed methods study. Participants were LifeSpan Coordinators, The Collaborative and working group members at four LifeSpan sites in New South Wales (three metropolitan/regional, one regional/rural). Quantitative social network data was collected through an online survey and analysed using Gephi software. Qualitative data through focus groups and interviews with Lifespan Coordinators and community stakeholders.

**Results:**

The social network survey was administered in three sites and was completed by 83 people. Data gave quantitative evidence of increased engagement across key stakeholders in each region who had not previously been working together. Nominations of other collaborators showed this network extended beyond the formal structures of The Collaborative. LifeSpan Coordinators were empirically identified as key players in the networks. Qualitative data was collected from 53 individuals (18 interviews and five focus groups) from across all sites. Participants identified benefits of this collaborative approach including greater capacity to run activities, better communication between groups, identification of “who’s who” locally, improvement in the integration of priorities, services and activities, and personal support for previously isolated members. LifeSpan Coordinators were key to the smooth running of The Collaborative. This may represent a risk to sustainability if they left. The collaboration model that suited metropolitan sites was difficult to sustain in rural sites, but gains were seen in better coordinated postvention efforts.

**Conclusion:**

LifeSpan Coordinators were noted to be exceptional people who magnified the benefits of collaboration. Geographic proximity was a potent driver of social capital. Initial engagement with local stakeholders was seen as essential but time-consuming work in the implementation phase. Coordinators reported this important work was not always acknowledged as part of their formal role.

**Supplementary Information:**

The online version contains supplementary material available at 10.1186/s13033-022-00524-z.

## Introduction

It is widely acknowledged that mental health services in Australia suffer from fragmentation and gaps between general and mental health, and social services, and suffers from a whole-of-community approach. As several authors have observed, addressing this fragmentation of the system has been a goal of mental health policy and strategies in Australia for over 20 years with limited evidence of impact [1, 2]. As well as formal health and social services (governed and funded by disparate federal, state, local, non-government, and private agencies [3]), the capacity of all frontline workers and community members to respond to a person experiencing suicidal distress is key to suicide prevention [4]. Vision 2030 for Mental Health and Suicide Prevention, developed by the National Mental Health Commission of Australia speaks of delivering mental services through “a unified system that takes a whole-of-community, whole-of-life and person-centred approach to mental health” and recommends that the design of mental health and wellbeing services start with local communities [5].

Many initiatives in Australia aim to address this fragmentation through a strategy of structured liaison between key services. Partners in Recovery Programs, for example, show the value of having a care coordinator, knowledgeable about available general and mental health, and social services to facilitate support of people with severe and persistent mental illness [6]. Brophy describes these coordinators as “boundary spanners” [7]. While each coordinator may develop their network of contacts through personal agency, their role is as a formal liaison across sectors. We argue that the key to successful implementation of mental health initiatives, and progress towards a wholistic response to need, is to prioritise a strategy of building social capital across key stakeholders in the community.

### Social capital

Social capital is succinctly defined as the “value in social networks” [8]. Networks provide social support, a sense of belonging, and allow access to practical assistance, resources, expertise, and experience. Social capital arising from the local community is recognised as having a positive influence on mental health [9]. Collaborative community networks or partnerships have long been recognised as valuable ways to address public health issues linking formal services and informal groups with a common interest or goal.

Health and community service networks aim to change two separate but related factors. Firstly, they aim to change the community context in which health behaviours of individuals are grounded. By changing the context and harnessing positive factors within the community (e.g., a sense of belonging and inclusion, providing access to practical support) improvements in health outcomes can be achieved [10]. Secondly, collaborative community networks seek to improve health outcomes through interventional programs. Collaboration between local service agencies focusses action by clarifying the vision or agenda of the community, allows access to varied expertise, experience and resources, and can greatly enhance the reach of the program through the larger cumulative network of collaborative members [11].

### The LifeSpan Initiative

The Black Dog Institute (BDI), a medical research institute focussed on all aspects of mental health, developed LifeSpan as a new evidence-based approach to suicide prevention, grounded in the community and harnessing lived experience [12, 13]. It is known that single interventions aimed at reducing the suicide rate have limited impact [14, 15]. Instead, a suite of interventions, covering a range of issues and targeting different groups is needed to effect change. LifeSpan combines nine different strategies into one program, each addressing a different population group or issue. Details of the LifeSpan program and the evidence behind individual strategies are given elsewhere [16] but briefly, the nine strategies are: improving emergency and follow up care for those in suicidal crisis; using evidence-based treatments; better equipping primary care to identify and support people in distress; improving the competency and confidence of frontline workers to deal with suicidal crisis; partnering with schools to promote help-seeking, mental health and resilience; engaging the community and providing opportunities to be part of the change; training the community to recognise and respond to suicidality; encouraging safe and purposeful media reporting; and improving safety and reducing access to means of suicide [16, 17]. Collectively, these strategies are intended to engage the whole community in the goal of suicide prevention and build capacity to identify and respond to suicide risk. The delivery of these strategies was managed at each implementation site by LifeSpan Coordinators in collaboration with the LifeSpan central team at BDI. Given these multiple strands within the program, robust collaboration with existing programs (e.g., TheWayBack, EveryMind, and Wellways), referral and communication networks between the LifeSpan staff and with stakeholders in the community, education, and health systems, are paramount to successful implementation [18]. The priority for LifeSpan Coordinators was to build social capital across the community in order to facilitate these processes.

Evidence of the impact of the implementation strategy of building social capital has been specifically reported in several studies. Communities that Care (CTC) projects that addressed mental health of young people in the UK and US and included the development of a cohesive local collaborative as a key strategy. They reported lack of success at some demonstration sites due to high turnover of staff, poor coordination, collaboration difficulties among professionals, and loss of local champions [9]. More recently, the optimised suicide prevention program (OSPI-Europe) invested in building local advisory groups across sites/countries and claimed much of the success of the interventions to that strategy of building social capital. They note the value of social capital in: (i) local partners with a positive track record being able to add to collective perceptions of feasibility of interventions and capability to deliver them within the advisory group, (ii) benefits of sector representatives facilitating access to participants (circumventing the barrier of gatekeepers); and personal rewards of expanding individual social and professional networks [18].

Recognising that social capital is a known facilitator of whole-of-community programs [9, 18], BDI considered it important to develop a robust collaboration and referral network among the different strands of LifeSpan, to link up resources and expertise, provide insight and appropriate access to priority populations, assist with recruitment of various agencies’ members for training, and to ensure clear, cohesive communication throughout each site’s region [17, 18]. Therefore, there was a key implementation strategy of convening an advisory group with broad representation to facilitate LifeSpan at each site. Each site was required to form this central group (or harness an existing group for this purpose) as part of their expression of interest to the BDI. LifeSpan Coordinators set engagement with this group and wider community engagement with local mental health services and other agencies and groups working in the suicide prevention field (e.g., local councils, non-government organisations, workplaces, schools) as a high priority. In this study, we consider changes around community social capital and connection of different local groups over time, as related to the LifeSpan program, to deal with the challenge of suicide prevention.

Social network research is a unique methodology that allows visualisation and quantification of the social context of a setting [19, 20] by mapping the extent and nature of relationships between members of a defined group. Relationships such as initiating collaborative partnerships, seeking advice, or learning complex new processes through mentorship are examples [21–25]. Sociograms and network parameters such as density can diagnose strengths of the group, and risks to the efficient operation of the network, and suggest specific interventions to strengthen network function [19]. Longitudinal data can be used to track growth or decay of ties and the effect of interventions. We hypothesised that sociograms, illustrating the links between key community stakeholders before and after the LifeSpan initiative would provide empirical evidence of the effect of this community strategy by showing the growth of collaboration between people working at each site in the suicide prevention field.

The aims of this study were to assess the existing networks between groups working in the suicide prevention space at baseline in each site (before the LifeSpan project started) and then to assess how these networks changed as a result of the implementation of LifeSpan. We equated the change in these networks with a change in social capital. The results of this study provide useful information not only in the context of LifeSpan, but for other similar community-based multilevel suicide prevention approaches, seeking engagement with the whole community.

## Methods

### Design

We used a mixed methods design for this study: quantitative data from an online social network survey, and qualitative data from a series of focus groups and interviews held at each of the four sites. Ethical approval for the study was granted by the Hunter New England Human Research Ethics Committee (2019/ETH03862).

### Setting

LifeSpan was implemented at four sites which were selected from an expression of interest process across regional and rural New South Wales, Australia. We refer to the sites by pseudonyms: Sites Alpha, Beta, Gamma and Delta. The current study was part of a larger implementation evaluation of the LifeSpan program. The model for implementation was similar but unique at each site with the Coordinators coming under the governance of either the Local Health District (LHD) that provide acute hospital and outreach community services, the Primary Health Network (PHN) that provide general practitioner and community based allied health services, or both. Three of the key activities that were undertaken at all sites were Youth Aware of Mental Health (YAM) in secondary schools [26], Question, Persuade, Refer (QPR) training on how to talk about mental health with colleagues or friends in the broader community, and local mental health services engagement. Table 1 provides basic information about each site.

**Table 1:** Description of the four sites (LHD = Local Health District; PHN = Primary Health Network)

Site	Hosted by	Setting	Interviews*	Number of Focus Groups (participants)
Site Alpha	LHD	Metro / Regional	3	1 (5)
Site Beta	PHN & LHD	Metro / Regional	3	3 (26)
Site Gamma	LHD	Metro / Regional	4*	1 (10)
Site Delta	PHN	Regional / Rural	6*	n/a

*The LifeSpan coordinators were interviewed twice at two different time points

At each site the LifeSpan Coordinators worked with the central collaborative group and convened a number of smaller working groups as part of the implementation process of Lifespan in their area. This was done differently at each site, although all sites utilised the existing mental health collaborations. The central collaborative group was referred to by different names at each site, e.g., “the Alliance.” We have standardised the terminology to “The Collaborative” across all sites (which includes the various associated LifeSpan working groups) for clarity in the reporting of our results.

LifeSpan was delivered via a stepped wedge design where sites were randomised to a staggered start date. The research team first engaged each site in July of 2019 in the final year of active implementation of the LifeSpan model. Two sites had completed active implementation at this point. Initial contact sought to establish cordial relations with site coordinators and establish a plan for evaluation activities going forward. All personnel across each respective site were made aware of an external evaluation of the implementation of the Lifespan program.

### Quantitative data collection: social network study

Data was collected through an online survey, which asked respondents about their socio-professional networks, prior to, and following, the implementation of LifeSpan in their area. Surveys were designed in consultation with LifeSpan Coordinators at each site to ensure clear wording, and to obtain membership lists of the working groups. All members of The Collaborative and/or working groups formed at three of the four sites (Alpha, Beta and Gamma) as well as their respective LifeSpan Coordinators, were identified and invited to participate. The survey was deemed inappropriate at Site Delta at that time due to a number of local issues (including lack of capacity to attend meetings, recent catastrophic bushfires, and COVID-19 response).

Individual surveys at each site were administered at least 12 months after LifeSpan activities had commenced to allow time for the network to be initiated. Potential participants were invited via an email containing survey information and a secure link. Where possible, the survey was completed at the end of focus groups or interviews to smooth the data collection process and minimise disruption for staff and stakeholders. A paper *Participant Information* form was made available for this purpose**.** All participants were required to provide consent on the landing page of the survey before progressing to the questions. The online survey platform used was *Qualtrics* [27]. Participants who accidently completed the survey twice were identified and duplicate data removed.

Respondents were presented with a list of people identified as LifeSpan Collaborative or working group members (see Additional file 1 for survey questions). For each name provided, respondents were asked if they were collaborating with the person, had made the collaborative link as a result of LifeSpan or whether it was a pre-existing link, and to specify the nature of the link (referral, shared care of a client, working in other ways). While names were used on the survey to reliably identify contacts and allow aggregation of data across respondents, once submitted, data were coded, and names were removed.

#### Social network analysis

Social network data was analysed using *UCInet v.6* [28] and diagrams of the relationships were constructed using *Gephi 0.9.2* software [29]. Network parameters of density, centrality and brokerage were computed. Social network parameters and definitions are shown in Table 2.

**Table 2:** Social network parameters and their definitions

Term	Definition
Node	Each node represents a member of the network
Tie	A tie represents a self-reported link between two nodes
Density	The number of actual ties divided by the number of possible ties. Reported as a percentage
Degree	Number of ties per node (either nominated by others or by the member themselves)
Indegree	Number of ties reported by others directed to the focal member
Centrality	Members with the highest interaction (ties to and from) with others
Betweenness centrality	Members who have high brokerage potential as they link two nodes that are not otherwise linked

### Qualitative data collection: interviews and focus groups

Qualitative data were collected through semi-structured interviews and focus groups at each site involving multiple stakeholders engaged in the implementation process in various organisational and community level positions. The researchers scheduled interviews with LifeSpan Coordinators at each site and aligned focus groups to coincide with regular collaborative meetings, to maximise participation. Key personnel involved in Lifespan activities locally were identified for interview in consultation with the LifeSpan team at BDI and the local LifeSpan Coordinators. All individuals received a consent form and information sheet outlining the nature of the research and the anonymity of their responses. Focus groups were conducted face to face while interviews were undertaken either in person or via Zoom, depending on availability. All interviews and focus groups were conducted by senior health services researchers experienced in qualitative research (YZ, JL, LE) and were audio recorded and transcribed verbatim.

Drawing on the Consolidated Framework of Implementation Research (CFIR)[30] (described in more detail below), an interview guide was developed in which questions acted as prompts allowing for the development of relevant issues as they emerged in both the face-to-face interviews and focus groups conversations [31]. Questions explored the fidelity of LifeSpan in each region, the barriers and enablers to implementation, insights into key roles associated with implementation, and how existing and new networks and/or relationships facilitated the delivery of Lifespan.

#### Qualitative analysis

All interview and focus group data were transcribed and imported into NVivo 12 for analysis [32]. Data analysis followed a thematic deductive approach utilising the CFIR as an analytical framework with inductive insights added as the coding process evolved. The CFIR is a comprehensive framework based on theory, that provides a way to systematically assess contextual factors influencing implementation of complex programs within complex contexts [30]*.* A key construct within this study was in the Inner Setting Domain: ‘Networks and Communications’. This construct facilitated description of the nature, quality, and evolution of organisational networks within the inner context. The Inner Setting was defined with respect to the organisational variables and factors that had direct influence on and involvement in LifeSpan implementation at each site.

The analytic work was an iterative process and was performed side by side with the data collection due to the time between conducting interviews. This allowed for the outcomes of initial coding to be fed back into subsequent interview analysis. A three-step process for coding the interview and focus group data was utilised to ensure intercoder reliability [33]. The initial stage involved researchers (JL, CR, LE & YZ) using the CFIR to independently code an interview transcript. This coding along with inductive insights that opened up other avenues of interest was then compared for reliability among the research team and any inconsistencies were discussed and agreed upon. This led to a high degree of intercoder reliability among the research team [34]. The final stage involved the coding of the remainder of the transcripts utilising the CFIR and following agreed upon inductive insights. Frequent meetings continued to maintain analytic rigour. Additional file 2 shows the COnsolidated criteria for REporting Qualitative research (COREQ) checklist for qualitative research [35].

## Results

### Social network survey

The response rate for the three site surveys ranged between 15 and 67%, (Table 3). At each site, we were confident that data from the key network players had been captured (justifying the seemingly low response rates). All respondents gave consent on the landing page.

**Table 3:** Survey responses by site

	Invited (n)	Respondents (n)	Response rate (%)
Site Alpha	103	26	25%
Site Beta	70	46	67%
Site Gamma	75	11	15%

**Fig. 1:**
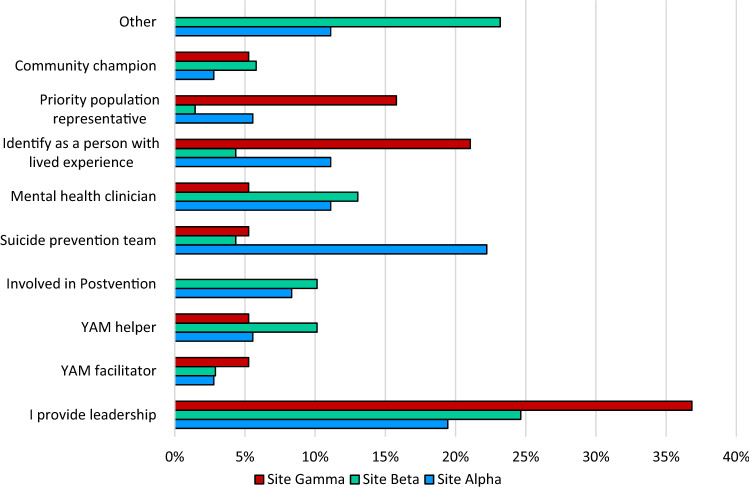
Percentage of respondents at each site nominating their role. Note respondents could select more than one role. (YAM = Youth Aware Mental Health)

Respondents were asked to select their role from a list of ten options. Selection of multiple roles was possible. The categories were: leadership, mental health clinician, YAM facilitator or helper, community champion, person with lived experience (community advocate), suicide prevention team, involved in postvention, priority population representative, and other. Roles added under the ‘other’ category included: Mental Health First Aid Officer, trainer, paramedic, and drug and alcohol worker. Most frequent response at Site Beta and Gamma was “I provide leadership,” and at Site Alpha, “Suicide prevention team.” Results are summarised in Fig. 1.

**Fig. 2:**
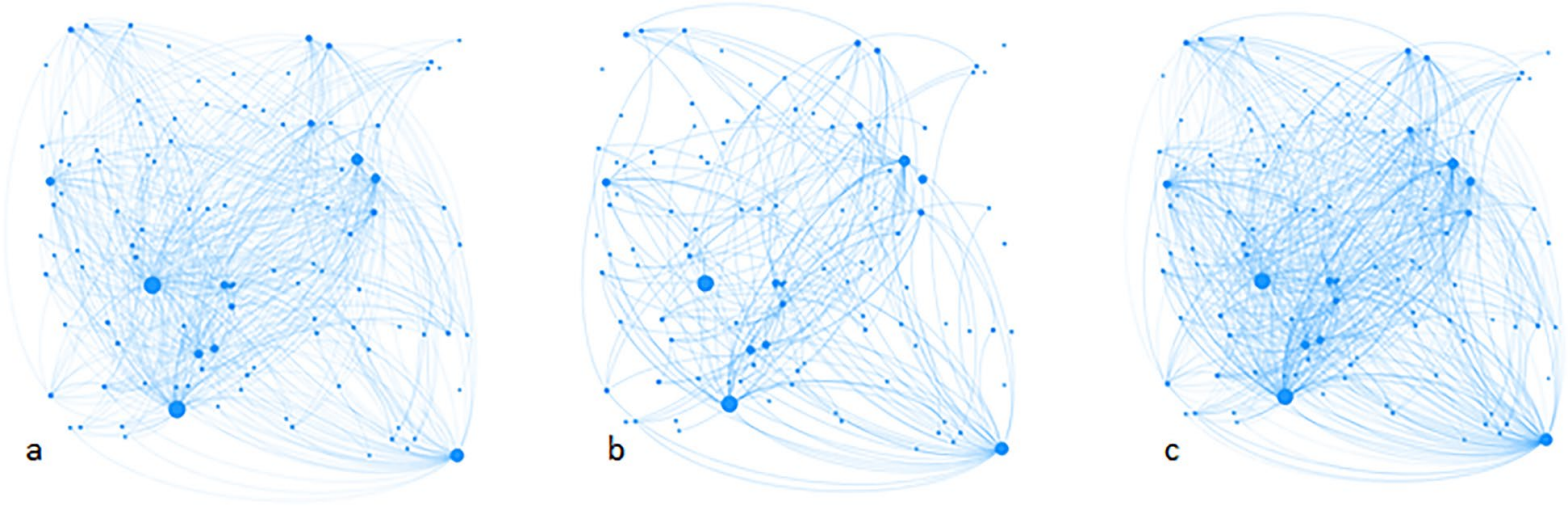
**a** Site Alpha ties to members that respondents knew before the LifeSpan intervention (existing ties), **b** Site Alpha ties to members that respondents only met through the LifeSpan intervention (new ties), **c** Site Alpha all ties. Each node represents a person. Lines joining nodes indicates a relationship (tie) reported by the respondents. Size of the nodes indicates their relative importance in the network (proportional to the number of times a respondent nominated them as a tie)

**Fig. 3:**
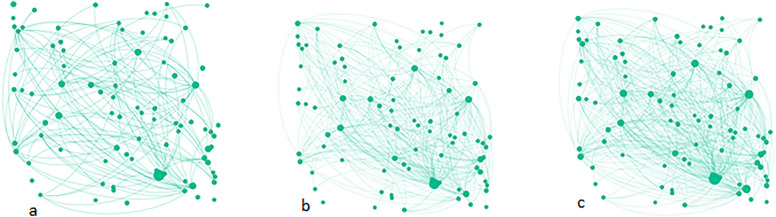
**a** Site Beta ties to members that respondents knew before the LifeSpan intervention (existing ties), **b** Site Beta ties to members that respondents only met through the LifeSpan intervention (new ties), **c** Site Beta all ties. Each node represents a person. Lines joining nodes indicates a relationship (ties) reported by the respondents. Size of the nodes indicates their relative importance in the network (proportional to the number of times a respondent nominated them as a tie)

**Fig. 4:**
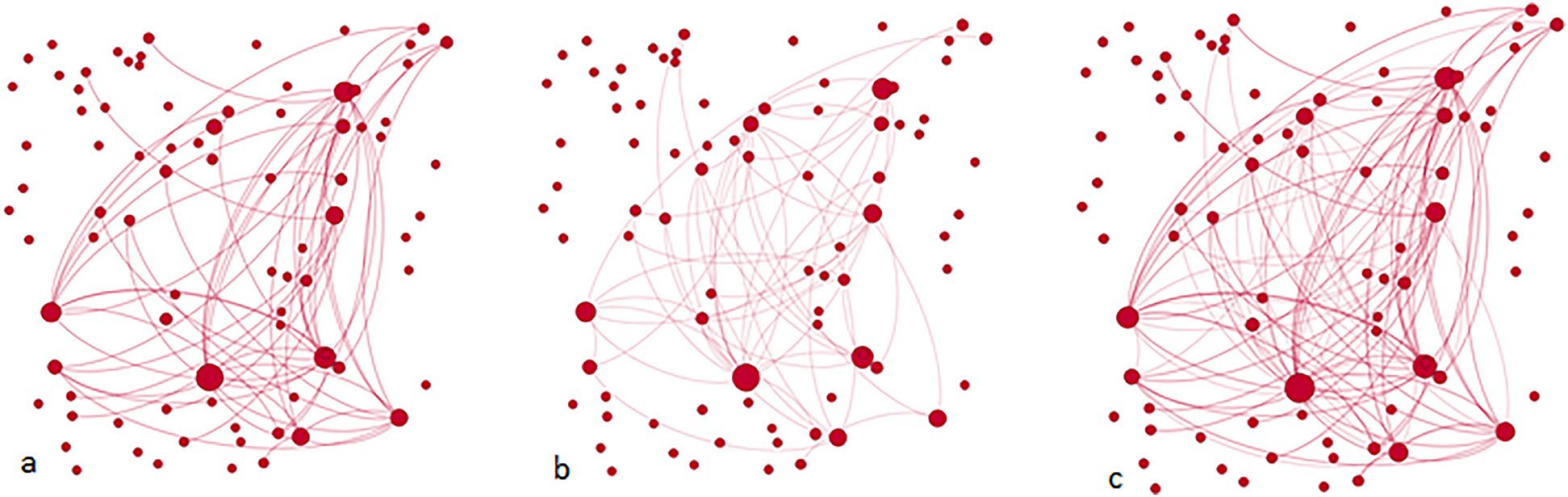
a Site Gamma ties to members that respondents knew before the LifeSpan intervention (existing ties), **b** Site Gamma ties to members that respondents only met through the LifeSpan intervention (new ties), **c** Site Gamma all ties. Each node represents a person. Lines joining nodes indicates a relationship reported by the respondents. Size of the nodes indicates their relative importance in the network (proportional to the number of times a respondent nominated them as a tie)

Three network diagrams are shown for each of the sites: Alpha, Beta and Gamma in Figs. 2, 3 and 4. The first shows existing ties from before LifeSpan, the second, new ties that came about through LifeSpan, and the third shows all ties at the time of the survey. Respondents at each site numbered 26, 46 and 12 (at Sites Alpha, Beta and Gamma respectively). Each respondent nominated an average of 5.2, 4.9 and 3.3 ties respectively, which included people from outside our list of identified members. The number of new ties at each site made up 71%, 70% and 47% of the total ties, (Table 4). People who were nominated the most (highest centrality) at each of the sites were the LifeSpan Coordinators. People with the highest brokerage potential (connecting two other people by the shortest path) tended to be people in executive / senior management or coordination roles of larger services; for example, local PHN, Aboriginal Health Service, LHD, or Lived Experience Group.

**Table 4:** Characteristics of the social networks of the three sites

Network metric	Site Alpha	Site Beta	Site Gamma
Density of collaboration network	0.05	0.10	0.10
Number of respondents reporting their ties	26	46	12
Number of people shown in the network	107	61	85
Number of ties reported (total)	536	438	108
Number of new ties to people they did not know pre-network (%)	380 (71%)	307 (70%)	51 (47%)
Number of people nominated from outside our list of identified collaborators	4	4	10
Average degree	5.2	4.9	3.3

#### Qualitative data

A total of 53 individuals responded to our invitation and participated in interviews and focus groups across the four sites. Eighteen interviews, some of which were in group format (2–3 people) due to time constraints, and five focus groups were conducted across all four sites (see Table 1). Only interviews were conducted at Site Delta. Interviews and focus groups mostly took place at the end of a scheduled LifeSpan Collaborative meeting, or via Zoom. Interviews and focus groups lasted around an hour.

Eleven subthemes that illustrate the CFIR concept “networks and communications” at the sites were identified from the interview and focus group data and are shown with exemplary quotes in Table 5. Roman numerals below refer to relevant quotes in Table 5 (e.g., Table 5: ii).

**Table 5:** Exemplary quotes from the focus groups and interviews undertaken with stakeholders involved with LifeSpan

	Theme/issue	Exemplar quote	Source
i	Value of the Collaborative approach	Facilitator: What do you think have been the key lessons that have come out of this part of the [LifeSpan] project? [LHD member]: I'd say having [a Collaborative] is central to achieving new goals… I think I'd have to own that when [LifeSpan Coordinators] said that they were going to approach the Chamber of Commerce and do some work with them, I thought here we go. This is a bit out there! But actually, it has been really valuable. That's put a lot of people into the QPR [Question, Persuade, Refer] process	Site Beta FG #1[LHD]
		Facilitator: What are the key lessons from [Site Alpha] that you would tell new sites about? [Priority Population health professional] I think definitely use the round table type [approach] … come together. I think that is really positive	Site Alpha FG#3 [Priority Population health professional 1]
		I think the value [of the Collaborative group] was acknowledged; that there was a big hole in this area. And this was only the beginning of the process. Trying to get people together and talking and seeing what we could offer	Site Beta FG #1 [Police]
ii	Inviting people to join the LifeSpan Collaborative was labour intensive at some sites	I did feel like I was spending a lot of time having coffees and talking to people and not a lot of time ‘working’	Site Delta FG#1 [LifeSpan Coordinator A]
iii	LifeSpan is the “anchor” or “glue” of the community effort	[LifeSpan coordinator] would attend all of those groups. I think they were sort of the one anchor that, they kept, even though we were autonomous and we were allowed to develop some stuff by ourselves, they kept us focused, even though we were also looking at what was happening more locally, on the ground for ourselves, you know	Site Alpha FG#3 [Priority Population health professional 1]
		But you can see how this [Collaborative group] kind of held a lot—no matter what happened, we were here together, working on it, trying to make the best of the situation, whether it was good, bad or whatever. So, you know, we are grateful to LifeSpan for helping be that glue	Site Gamma FG#1 [LHD]
iv	Value of a community approach to activities	Going back a number of years, there was a lot of activity [after a death by suicide], but it really lacked that coordination. I don't think anyone was taking responsibility for identification of when there's been a death, who needs to be involved in that response, is there someone already involved?	Site Delta [PHN]
v	Value of support within the Collaborative	Certainly, from our perspective, we just see too much of it [death by suicide] every day. We had one yesterday, with one of our ex colleagues. Yeah, there really has been a lack in support for families as well as the individuals. So, to get all the players together to a one stop shop is a great idea. And it's the starting point. It's not the end point. You can only keep going from there	Site Beta FG #1 [Police]
vi	Achieving and maintaining buy-in	I sit on the executive [of the Collaborative group] and we want everybody in the [group] to have buy-in. However, you're right, at the executive level, we do talk about things and we say 'okay, well we've made the decision, but that's not final. Now we need to take it back to the group today.' And so yeah, it does take that little bit longer, but it has that impact of everyone has that buy-in. … By definition, it is a Collaborative. It is astonishing in my view to see how much work and the goodwill that gets done under that	Site Alpha FG#3 [LifeLine]
vii	Linking people to resources/help	Setting up the website, all those kinds of things that we're linking people in the community it's probably been one of the most valuable and particularly in engaging businesses… That's the feedback I keep getting when I travel around is people in businesses saying that we didn't know; or we are still finding businesses that are saying where do we go to get help? And we can direct them to that help	Site Beta FG#1 [LifeSpan Coordinator]
		It is good to know that the players, like getting a group together who actually know the individual players in each area to speak over issues or put people in touch with individuals	Site Beta FG #1 [Police]
viii	Flat hierarchy	I find that the networking aspects of [the Collaborative] is really important. And I love the way that usually that leadership, I love the way that anyone can be invited to champion this cause regardless of whether you're in a high level, executive position, or, you know, if you're just a regular person	Site Alpha Focus Group #1 [Priority population Health professional 2]
ix	Integrating care; aligning of objectives	How does health and the PHN work together? I think that is an important component of it. I think that has been really helpful having [LHD member] and then now [PHN member] on board. But before that we were kind of playing catch up all the time between the two services about where we were going, and the idea of stepped care and all those things	Site Beta FG [PHN]
x	“Mapping” of key services	What I've noticed is that people wanted to do something, and that it's disparate. When I first started, there were disparate groups all over the place, not coming together. And saying:*“Well, we do that bit”*, *“Oh, I never knew you did that. Oh, I thought you did this”*, *“No, no, we don't do that.”* And then you are finding out, it's basically a bit of a mapping of who are the key movers and shakers in our region? Who’s actually doing prevention, early Intervention, critical intervention and postvention. And we’re getting that clearer now with the [Collaborative] members	Site Beta Interview [LifeSpan Coordinator]
xi	Success dependent on geography to some extent	The way that we set up our [Collaborative] was based off a metro model and has never really gotten the same traction as the metro [Collaboratives] …, but in saying that, we’ve had some individuals or working groups that have helped implement those strategies anyway, but the one big meeting to share knowledge and, you know, implement things just never worked	Site Delta FG#1 [LifeSpan Coordinator A]
		It's one of the advantages of not being a group of suburbs. That this is a town and people know each other in [Site Gamma]. Everybody's got a connection to somebody, somewhere and if you need or want anything, someone will know who to talk to	Site Gamma FG#1 [LHD 2]
xii	Building on existing Collaborative relationships	I think we had really good local leadership and commitment [at the start of the LifeSpan project], as evidenced by all the people who came to the table regularly. And that was really built on a bedrock of good work that’s been done in this area for a long time. So, it wasn't like we were manufacturing it out of nothing	Site Gamma FG#1 [LHD 1]
		There's a sense of community spirit [in Site Gamma]. I can see it. I've been here 12 years. I can see it amongst families and communities… you can see that basic social connection. Local intel, isn't it?	Site Gamma FG#1 [LHD 2
xiii	LifeSpan Coordinators’ role in building social capital	One of my standout things around [the LifeSpan Coordinators] aside from the support that they give us, it's their genuine, and I really mean that, belief in what people with a lived experience can offer… sitting around a table with people who might have degrees as long as their arms and are called doctors and the CEOs. And to know that what you have to say, is just as important to them, as what they have to say is important to us, that is a big, big plus	Site Alpha_FG#2 [Person with lived experience]
		I actually started … having a bit of a vent, and I actually did get quite emotional, as I do. And you know, they rang me and [LifeSpan Coordinator] was like 'are you okay?' and so that checking in stuff as well. So, you know, we're not just collaborative members, we are people and we are, you know, we feel pain and sadness and tiredness all that sort of stuff, and I think, yeah, [the LifeSpan Coordinators] have really, it's still personal, and they still treat us like people and that's lovely sometimes when you see, you know, collaborative members around town or you know at council or wherever you are different places and everyone was like, 'Oh how are you going?' you know, so yeah, again I think it's more than collaborative, it's friendships, it's relationships and I think that's the thing isn't it, it's that relationship	Site Alpha FG#3 [Priority population health professional 1]
xiv	LifeSpan Coordinators seen as knowledge brokers	I think the things that the coordinator role provided was—it was a bit of an umbrella view of the local suicide prevention work that was occurring … Because everyone knows the bit that is happening in relation to them and their organization, but don't know all the other stuff. And that’s really important … so you don’t duplicate and can share resources, you know. And that's the sort of role that doesn't exist… I think it was that umbrella view and being able to connect people, resources and information. And so I think you have to define that as acting as a bit of a knowledge broker as well. We would become the people that the people would ring with anything to do with suicide and suicide prevention, really, anything locally – including, clinical inquiries, which we were really not the people to deal with that, including being told about when postvention responses were being coordinated. You know we were the go to, but the role we could play in that was to connect the right people, or finding out who the right people were, because we had a really big network of contacts	Site Gamma Interview [LifeSpan Coordinator]

*LHD* Local Health District, *PHN* Primary Health Network, *FG* Focus Group

The process of forming or enhancing an existing Collaborative group to work on LifeSpan projects was discussed by all interview and focus group participants within their respective sites, usually in the context of the question “What has worked well?” All LifeSpan Coordinators noted how their initial and ongoing priority for the program was identifying, meeting with, and inviting various stakeholders to collaborate with other local community groups and services that were already working in the local suicide prevention field. This ‘round table’ approach was agreed as a key strategy of the LifeSpan model that contributed to success (i).

At all sites, LifeSpan Coordinators noted that this work of engagement with different stakeholders took up a considerable amount of their time, at least initially. At one site, a Coordinator described a mismatch in expectations, as the work of engaging stakeholders was largely invisible to their hosting organisation managers (ii). Adding to this work, LifeSpan Coordinators invested a lot of time in keeping groups engaged and on task. A focus group participant likened a LifeSpan Coordinator to the anchor of the Collaborative group maintaining focus on projects at Site Alpha, while another participant called LifeSpan Coordinators “the glue” of the Collaborative groups when things were difficult (Site Gamma) (iii).

Terms of reference and operating rules for the different versions of The Collaborative were evident across sites. However, at every site, The Collaborative was built on existing relationships and this was seen as a key success factor (xii) that provided a strong foundation to support network development. Participants discussed the benefits of linking up several groups, that were previously isolated, to do a combined event. One notable community outcome of the linking of disparate service providers in the LifeSpan Collaborative was an improvement in the suicide postvention process at Site Delta. Prior to LifeSpan, it was not always known when a death by suicide had occurred or who had already responded. First responders (ambulance /police) to a death by suicide were now starting to directly inform key members of The Collaborative who could then mobilise postvention support teams (iv).

The value of The Collaborative group in linking themselves and the stakeholders they represented into a supportive network was discussed in several focus groups. Representatives from schools, the media, police, justice, commerce, priority population advocacy groups, and people with lived experience all expressed positive outcomes from being linked via The Collaborative into training and supportive expertise. Even organisations that already had formal support and training programs appreciated the additional resources (v). Several participants noted the value of LifeSpan Coordinators as people who could direct them to the appropriate resource or answer questions (vi). LifeSpan Coordinators were also seen as enablers, being inclusive and supporting grass-roots activities (vii). It was noted at some sites that LifeSpan, or more correctly the Collaborative group brought together by LifeSpan, had brought primary health and hospital services into step, moving towards better integrated care and alignment of priorities (viii).

A common theme within the sites was that several disparate and disconnected groups were working in the suicide prevention field across each region before the implementation of LifeSpan. At Site Beta and Site Delta, the LifeSpan Coordinators spoke at length about how fragmented the local suicide prevention effort was and how there was poor understanding of individual roles and visions among them collectively (x). LifeSpan activities, where these groups were identified and engaged was seen as important in building their capacity to make change.

At the rural Site Delta, geography was discussed at length in terms of the difficulties it posed to Collaborative working and integrated effort. The Collaborative group was not as successful as they would have liked as it was “based on the metro model”. The initial engagement of stakeholders in Site Delta also took longer and involved greater effort (e.g., driving for three hours one way to attend a requested face-to-face meeting) (xi).

The benefits that came from the increase in social capital were often specifically attributed to the work and qualities of the LifeSpan Coordinators who were praised for their approachability, passion, and integrity. Qualitative data collected from The Collaborative members consistently showed that the LifeSpan Coordinators were exceptional people who all were fully invested in their community. There was frequent praise from participants for the LifeSpan Coordinators’ openness to questions, follow-up phone calls, and for their supportiveness (xiii). Reflecting on their role in the suicide prevention field in their region, one LifeSpan Coordinator used the term “knowledge broker”, and explained how, because they had an “umbrella view” of all that was going on in the site they had become the go-to people to find help for problems or issues. This is reflected strongly in the quantitative social network analysis where LifeSpan Coordinators were identified as key brokers at their sites (xiv).

## Discussion

The findings from this study support that the social capital of local suicide prevention agencies was strategically increased as a result of having a collaborative network in each site, and was identified as a key success factor for the implementation of LifeSpan. Our evaluation of this aspect of the program clearly shows a positive change in the social structure of the communities that implemented LifeSpan in four areas of NSW. Our quantitative, social network data from three of the sites shows how collaborative links between community stakeholders increased after 12–24 months of implementation of LifeSpan. Qualitative data from the interviews and focus groups from all sites provide insight into how this change was achieved and the benefits that this increase in social capital brought both the community and individual members of the groups.

The strategy of building local social capital was unequivocally seen as positive and a key success factor of LifeSpan. The strategy of identifying, engaging and working with a Collaborative of local individuals and groups was seen as a core strength. It was frequently acknowledged that local groups working in suicide prevention were siloed and often unaware of other activities going on. This is consistent with the wealth of evidence that describes suicide prevention initiatives [4] and health services more broadly [3], as being fragmented in Australia. Through the Collaborative, there were opportunities to coordinate and share effort more productively.

Another manifestation of increased social capital was the effect of knowledge brokering through The Collaborative. Even for organisations with formal training or support services, knowing who to contact in The Collaborative for advice or support was highly valued by the participants. At Site Delta we heard in detail how the postvention response to a death by suicide was now improving as it was initiated and coordinated through The Collaborative network relationships. The LifeSpan Coordinator role as a broker was similar to Support Facilitators in other initiatives seeking to broker access and information across siloed services (e.g., [1, 2, 36]). This study provides additional evidence of the effectiveness of the role.

The Collaborative and working group members covered a broad range of community and organisational stakeholders, demonstrating a comprehensive whole-of-community approach [4]. There was representation from NSW Health (hospital, public Community Mental Health services, Drug and Alcohol Community teams), NGO mental health providers (e.g., WellWays, headspace, LifeLine), education (public schools), front-line responders (e.g., police, ambulance), people with lived experience, and priority population groups (e.g., First Nation people, LGBTQ +). At some sites extra stakeholder groups involved in suicide prevention were represented including private schools, coroners’ court, media outlets, and local church support groups. While not all groups were equally invested, and there was attrition and churn of members over time, this shows the success of the engagement strategy in terms of reach and a commitment to inclusivity.

The most notable group that failed to respond to the call to be involved was the clinical mental health workforce working in hospitals, and community health facilities, most apparent in Site Gamma. This lack of engagement can be understood in the context of constantly high workloads, continuous change, and clashing priorities with other mental health initiatives already underway leading to “change fatigue” and lack of capacity among mental health staff [37, 38]. While these are frequently reported barriers to change, we note that with limited time and nine separate interventions, LifeSpan Co-ordinators were more likely to concentrate on interventions in sectors and agencies that were facilitating access, e.g., schools. A separate paper is in preparation that will explore the barriers to engagement more fully but briefly, we recommend longer term strategies for engaging the clinical mental health workforce including explicit Executive sponsorship of LifeSpan, which often enables quarantined time for the project and alignment with other hospital or service priorities.

A large percentage of respondents across the sites chose “I provide leadership” as their role. This was paired with a wide range of second choices such as “person with a lived experience of suicide”, “YAM facilitator or helper,” “mental health clinician or “involved in postvention.” This confirmed that the people taking part in the Collaboratives at each site were key players in their respective areas and saw themselves as advocating and making a change on behalf of the group they represented. The OSPI-Europe project also found that Advisory Group members were key stakeholders and leaders of their respective groups facilitating access to participants for the interventions and helping with the dissemination of the work [18].

The process of identifying and engaging key stakeholders at each site was described as labour intensive and often “invisible” work which almost always fell to the LifeSpan Coordinators. This was experienced despite all of the LifeSpan Coordinators (except one, at Site Delta) being local residents, embedded in these regions and often well-known in the local mental health field. This presumably would give them a head start in identifying, accessing, and engaging with key stakeholders. Building networks as a local and “one of us”, rather than as an outsider, and having a known track record in the field is known to increase trust, an important precursor to engagement [39]. Whole-of-community approaches to suicide prevention [4] must factor in the time that this network building activity takes if any real integration and inclusion of all stakeholders is to be realised.

Different starting points contributed to the variation in scope and extent of network development across the sites. In areas where mental health services were siloed or fragmented, or where a culture of collaboration between community groups, NGO providers and NSW Health providers were limited before LifeSpan implementation, the development of functioning collaboratives was less extensive.

Sites expressing interest to host LifeSpan were required to convene or harness an existing collaborative group with wide representation from services, community groups, people with lived experience, and high priority populations. The fact that all the sites reported here had an existing collaborative group meant that LifeSpan was building on the social capital already existent at the site. LifeSpan activities none-the-less changed the social capital of the group increasing individual relationships, strengthening access to target populations and interagency networks. This suggests that sites that do not have a suicide prevention collaborative of this sort may benefit more from the establishment of a group as part of an implementation of LifeSpan, but that time to convene the group and form initial relationships, trust and understanding of roles would be necessary.

Although all implementation strategies and frameworks acknowledge the importance of engaging stakeholders (e.g., CFIR Process Construct [30]), the time it takes to do it effectively is likely underestimated. Some problems arose as a result of this with managers who were expecting activities to start quickly without a lengthy engagement period. Many of the LifeSpan Coordinators mentioned the value of face-to-face contact so they could learn about the person’s service and role and extend a personal invitation to join the LifeSpan Collaborative. At the rural Site Delta, the preference for face-to-face meetings meant that LifeSpan Coordinators would spend hours travelling to their appointments. It also meant that once the Collaborative had been established, in-person attendance at meetings were far less likely due to distance.

Site Delta did not take part in the social network survey. Although initial contact and engagement with stakeholders across the region was successful in the first period of implementation, changes in local LifeSpan personnel, and conflict with other local priorities (e.g., aftermath of catastrophic bushfires earlier in the year) meant that regional engagement with LifeSpan weakened over time. This was mentioned by all six interviewees. More importantly, we would argue, the vast distances between stakeholders and unstable internet service in Site Delta, meant this was not unexpected. Geographic proximity is a known driver of collaboration [40] mediated by face-to-face contact and chance local meetings (so called “corridor or car park” conversations). This supports the Site Delta LifeSpan Coordinator’s assertion that the “metro model” does not suit a geographically dispersed site. A smaller scale version of this geographic isolation was voiced by Collaborative members from outlying towns of the other Sites.

## Strengths and limitations

Social network research is uniquely placed to map the social context of a setting. Data from the social network survey was self-reported, which is a limitation, but the study design mitigated the risk of unreliable information. (i) The use of the roster format minimised recall bias ([41] p.48), (ii) the on-line mode, straightforward questions, and the assurance of anonymity reduced any skew due to social desirability [42].

While efforts were made to maximise the response rate at each site, this was not possible at Site Delta. This limits our evaluation of the only rural site to findings from the interviews. Further, networks are dynamic and so the sociograms are acknowledged to be a depiction of the networks a single point in time.

There are two main considerations in determining whether a whole network survey has an adequate response rate or not: the type of analysis being performed on the data (as some measures are more robust in the face of missing data than others [43]), and the suspected structure of the network. We are confident that we captured the most influential members whose high number of ties contribute most to the structure of the network [42]. We have also chosen robust analytics to avoid inappropriate interpretations of the data. An integrating synthesis of quantitative data from the social network survey and qualitative data from interviews and focus groups, provides a more holistic interpretation and is a strength of the paper (Additional files 1, 2).

## Conclusions

The LifeSpan suicide prevention program had a clear priority to identify and engage local groups and service providers to build the social capital of the community. All four sites showed a broad range of stakeholder groups that joined The Collaborative group and working groups. Clear benefits resulted from this: greater capacity to run activities, better communication between groups, identification of “who’s who” locally, improvement in the integration of priorities, services and activities, and personal support for previously isolated members. These benefits were often specifically attributed to the LifeSpan Coordinators who were praised for their approachability, passion, and integrity. The study has also revealed the limitations of this collaborative approach in geographically dispersed regions, where the potent driver of collaboration (in-person meetings) is precluded.

## Supplementary Information

**Additional file 1. **Social Network Survey.

**Additional file 2. **Consolidated criteria for reporting qualitative studies (COREQ): 32-item checklist.

## Data Availability

Data is not publicly available due to Ethics requirements that participants and sites remain un-identifiable.

## Abbreviations

CFIR: Consolidated Framework for Implementation Research
COREQ: COnsolidated criteria for REporting Qualitative research
CTC: Communities that Care
LHD: Local Health District
OSPI: Optimised suicide prevention program
PHN: Primary Health Network
QPR: Question, Persuade, Refer
YAM: Youth Aware of Mental Health
