# Supplementary material for: Networks to strengthen community social capital for suicide prevention in regional Australia: the LifeSpan suicide prevention initiative
Source: Int J Ment Health Syst. 2022 Feb 7;16:10. doi: 10.1186/s13033-022-00524-z (PMC8822835; doi:10.1186/s13033-022-00524-z)
Supplement: Supplementary file 1 — Additional file 1. Social Network Survey. [file 13033_2022_524_MOESM1_ESM.docx]

## Supplementary File A

Social Network Survey

Welcome to the [region name] LifeSpan / Suicide Prevention group Survey

You have received this invitation as you have been identified as someone working on a LifeSpan program activity and/or are working on a suicide prevention activity with the [region name suicide prevention group].

This survey has been authorised by the Black Dog Institute and is an important part of a process evaluation of LifeSpan.

The survey will take around 5-10 minutes to complete.

The survey will help us to understand how collaboration, communication and networks are configured in your local area and how LifeSpan/the [local suicide prevention group] have changed these. This information will allow us to gain a snapshot of [the region’s] networks working in the suicide prevention field and assess the impact of LifeSpan activities.

This survey includes a social network component. This maps the relationships between participants by surveying all members and then combining their answers.

We need to use names on the survey in order to construct each person’s web of contacts, however once submitted, data will be coded and de-identified. This is standard methodology for social network studies and has Ethics Committee approval. An example of a similar study is provided below.*


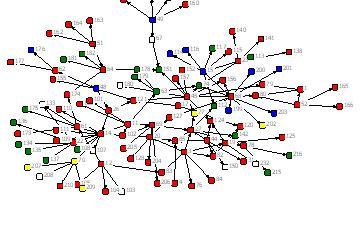

Each person is represented as a node and lines show if they know each other. Colours can show attributes of the members. In this map they show which health district people work in.

All individuals and institutions will be anonymous in all reporting of results. De-identified results of this survey will be made available to the LifeSpan executive for general dissemination.

This activity forms part of a project evaluation of LifeSpan undertaken by Assoc Prof Yvonne Zurynski, and Drs Louise Ellis and Janet Long from Macquarie University. This research has received Ethics approval from HNELHD HREC 2019/ETH03862. This survey is being conducted by Dr Janet Long, Research Fellow at Macquarie University. If you have any questions, please contact her by email: janet.long@mq.edu.au

The survey will remain open for the next week if you wish to complete it later.

*Long, J. C., et al. (2014). "Patterns of collaboration in complex networks: the example of a translational research network." BMC Health Services Research 14(1): 225. 

Are you happy to proceed?

Yes/ No [end of survey]

1. Firstly, what is your name and role? (This is needed to assign you a unique, anonymous code for the map of your contacts)

*Remember, all individuals and institutions will be anonymous in all analysis and reporting of results.*

e.g., John Doe, YAM facilitator, MyTown High School

1. Thinking about **your work in the suicide prevention field** [Region name] area over the last two years, how would you describe your role (select all that apply):

- I provide leadership
- YAM facilitator
- YAM helper
- Involved in Postvention
- Suicide prevention team
- Mental health clinician
- Identify as a person with lived experience (directly, as a carer or as a person bereaved by suicide)
- Priority population representative e.g. Aboriginal or Torres Strait Islander person
- Community champion
- Other __________________________________________

1. Do you work directly with consumers/patients/students?

Y/N

1. **Which of the groups** listed below have you been formally involved with (e.g., part of the planning or implementation team)? Please select all that apply.

- [Regional Schools group]
- [Regional Means restriction Group]
- Lived Experience
- [Regional LGBTIQ+ group]
- [Regional Aboriginal Community]
- [Regional CALD group]
- Black Dog Institute team

1. You have selected << Group X>> and <<Group Y>> [from Q4] ….

Below you will find a list of the **key people involved with each project**. Work your way down the list of people and for each one please select the appropriate statement.

*Example given here if they choose Lived experience I don’t know this person is the default answer.*

| **Lived experience group members** | **I don’t know this person** | **I only met this person through this project** | **I have worked with this person before** | **This is me** |
| --- | --- | --- | --- | --- |
| **[Group member #1]** |  |  |  |  |
| **[Group member #2]** |  |  |  |  |
| **[Group member #3]** |  |  |  |  |
| **[Group member #4]** |  |  |  |  |
| **[Group member #5]** |  |  |  |  |
| **[Group member #6]** |  |  |  |  |

*[Repeat Table of group members for each of the projects they say they are involved with].*

1. Have you been involved in any other projects **in the last 2 years** not listed above that are also in the suicide prevention field?

Yes / No

If yes: Please name them here (please include where they are being run).

______________________________________________________________________

_______________________________________________________________________

_______________________________________________________________________

_____________________________________________________________________

*[All respondents]*

1. It has been frequently observed that one of the benefits of the LifeSpan Project [in this region] is that since July 2017, people **have started to collaborate** with different groups, **make new contacts** and to **build the local suicide prevention network**. Would you agree with this observation?

Yes / No

*[If yes…]*

1. Please list any of your **new contacts** since the start of LifeSpan that you consider to be significant. There is room for **up to** 10 contacts. There is room below to add some comments about what you have written, if needed.

| Person | Role | Organisation |
| --- | --- | --- |
| e.g., John Doe | Head teacher | My Town High School |
|  |  |  |
|  |  |  |
|  |  |  |
|  |  |  |
|  |  |  |
|  |  |  |
|  |  |  |
|  |  |  |
|  |  |  |
|  |  |  |

Comments on new contacts list: ­­­­­­­­­­­­­­­­­­­­­­­­­­­­­­____________________________________________________________________________________________________________________________________________________________________

*[All respondents]*

1. Have you had contact with anyone form the other four LifeSpan sites (Yes/No)

*[If yes…]*

Which site/s?

- - [Site 1]
  - [Site 2]
  - [Site 3]

1. Are there any comments you would like to make about the LifeSpan program as run in the [local region]?

______________________________________________________________________________

______________________________________________________________________________

_______________________________________________________________________________

_______________________________________________________________________________

Q.10 Do you have any other comments or observations about suicide prevention activities more generally in the [local region]?

______________________________________________________________________________

______________________________________________________________________________

_______________________________________________________________________________

_______________________________________________________________________________

Thank you for your time. Your responses have been recorded.
